# Intimate partner violence among adolescents and young women: prevalence and associated factors in nine countries: a cross-sectional study

**DOI:** 10.1186/1471-2458-14-751

**Published:** 2014-07-25

**Authors:** Heidi Stöckl, Laura March, Christina Pallitto, Claudia Garcia-Moreno, on behalf of the WHO Multi-country Study team

**Affiliations:** Department of Global Health and Development, Gender Violence & Health Centre, London School of Hygiene and Tropical Medicine, 15-17 Tavistock Place, London, WC1H 9SH UK; INSERM, U912 (SESSTIM), 23 rue Stanislas Torrents, 13006 Marseille, France; Aix Marseille University, IRD, UMR-S912 Marseille, France; ORS PACA, Observatoire Régional de la Santé Provence Alpes Côte d’Azur, Marseille, France; Department of Reproductive Health and Research, World Health Organisation, Avenue Appia 20, 1202 Geneva, Switzerland; Department of Reproductive Health and Research, Reproductive Rights and Adolescence, World Health Organisation, Avenue Appia 20, 1202 Geneva, Switzerland

**Keywords:** Intimate partner violence, Prevalence, Risk factors, Adolescents, Young women, Population-based survey

## Abstract

**Background:**

Little is known about the prevalence of intimate partner violence (IPV) and its associated factors among adolescents and younger women.

**Methods:**

This study analyzed data from nine countries of the WHO Multi-country Study on Women’s Health and Domestic Violence against Women, a population based survey conducted in ten countries between 2000 and 2004.

**Results:**

The lifetime prevalence of IPV ranged from 19 to 66 percent among women aged 15 to 24, with most sites reporting prevalence above 50 percent. Factors significantly associated with IPV across most sites included witnessing violence against the mother, partner’s heavy drinking and involvement in fights, women’s experience of unwanted first sex, frequent quarrels and partner’s controlling behavior. Adolescent and young women face a substantially higher risk of experiencing IPV than older women.

**Conclusion:**

Adolescence and early adulthood is an important period in laying the foundation for healthy and stable relationships, and women’s health and well-being overall. Ensuring that adolescents and young women enjoy relationships free of violence is an important investment in their future.

## Background

Violence against women, especially by an intimate partner is receiving increased attention due to its widespread nature and severe health consequences [1, 2]. The WHO Multi-country Study on Women’s Health and Domestic Violence against women, conducted between 2000 and 2004 found that between 13 and 61 percent of ever-partnered women aged 15 to 49 years in 10 different countries across the world reported having ever experienced physical or sexual violence from their partner [3]. The prevalence of intimate partner violence (IPV) is assumed to be even higher among adolescents and young women, a claim that is sustained by evidence from the USA, where the majority of studies on IPV among adolescents and young adults originate from [4–8]. However, evidence increasingly emerges from non-industrial countries on the prevalence, risk factors and health effects of IPV among this population [9, 10], especially among student populations [11, 12] and in respect to the high risk posed by early marriage [13–15].

Women’s physical health is often relatively high in most settings during adolescence and early adulthood. At the same time, adolescence and early adulthood is a time of rapid physical, psychological and cognitive changes, stress and experimentation, which can be psychologically taxing and often overwhelming [9]. Causes of mortality in later life stages often have their origin in events or behaviors developed during adolescence [16] as adolescents and young adults are most likely to engage in risky and unhealthy behavior, such as substance abuse, school dropout, eating disorders, high-risk sexual behaviors, lack of physical activity and early pregnancy. IPV might up to double adolescents and young adults’ likelihood to engage in those types of risky behavior [9, 17–21]. Studies in sub-Saharan Africa and India have established that experiences of IPV and sexual coercion increases young women’s risk of HIV infection, regardless of whether they occur in an early marriage or a dating relationship [10, 13, 14, 22]. IPV in adolescence and young adulthood has also been found to negatively impact young women’s educational attainments in non-industrialized countries, with many women leaving school upon marriage [14, 15].

Adolescence and young adulthood is a crucial period for establishing the foundations for women’s future health and life [23, 24]. The effect of experiencing IPV at this period in life is likely to affect the physical and psychological as well as the economic well-being of adolescents and young adults in the future. Investing in a life free of violence for adolescents and young women therefore may be an important investment in their future [25].

To design preventive strategies to promote healthy relationships and developmental outcomes, as well as responses for adolescents and young women who experience IPV, more evidence is needed on what factors in young women’s lives put them at increased risk of IPV [26]. Existing studies found that IPV during adolescence is associated with witnessing or experiencing IPV during childhood, atypical family structures, multi-partnering, and substance, abuse, especially alcohol abuse [6, 9, 17, 18, 27, 28]. However, most of these studies were conducted in the US and are not necessarily generalizable to other contexts. Studies conducted in non-industrialized countries highlight the increased risk of economic hardship and early marriage on adolescents or young women’s experience of IPV or sexual coercion in marital or dating relationships [10–13]. It remains difficult to compare the findings across these studies, as they differ in terms of measurement and sampling strategies, age groups interviewed and study objectives. There clearly is a paucity of population based studies on IPV among adolescent and young women, with many studies being limited to samples of college and high school students [5, 18], which are not representative of the general population of adolescents and young adults.

While there are studies investigating the prevalence, risk and associated factors for IPV among women of reproductive age across industrialized, middle and low income countries [29], none focused exclusively on adolescent and young women. This group, however, deserves special attention as IPV has been found to be more prevalent in young age and because of its potential knock-on effect on later experiences of violence, poor health and social capital [6, 9, 11]. This study seeks to close this gap by estimating the prevalence of IPV among women aged 15 to 24 and to explore the risk factors associated with it in nine countries and 14 sites, by analyzing data from the WHO Multi-country Study on Women’s Health and Domestic Violence against Women.

## Method

The WHO Multi-country Study on Women’s Health and Domestic Violence against Women surveyed over 24,000 women in 10 countries between 2000 and 2004. Details of the study have been reported elsewhere [30]. Briefly, the study collected population-based, quantitative data on IPV among women aged 15–49 years, using the same study design, face-to-face interviews of the same questionnaire, female interviewer training and procedures across all sites. Study sites included Bangladesh, Brazil, Ethiopia, Japan, Namibia, Peru, the United Republic of Tanzania, Samoa, Serbia, and Thailand. Japan was excluded from this analysis since women below the age of 18 could not be interviewed. If possible, surveys were conducted in a rural and urban site in each country.

### IPV outcome

Women were considered to have experienced physical and/or sexual violence by an intimate partner if they reported experiencing at least one specific act by a current or former intimate partner since the age of 15. Specific acts of physical violence include being slapped or had something thrown at her, being pushed or shoved, being hit with a fist or something that could have hurt, being kicked, dragged or beaten up, being choked or burnt on purpose or being threatened or hurt with a gun, knife or weapon. Specific acts of sexual violence are being physically forced to have sexual intercourse, having had unwanted sexual intercourse because of fear of what the partner might do and being forced to do something sexual that she found degrading or humiliating. Women who reported experiencing one of these acts of physical or sexual violence, which had the answer options yes and no, by their partner were asked if this had occurred in the last year or ever.

### Conceptual framework

**Figure 1:**
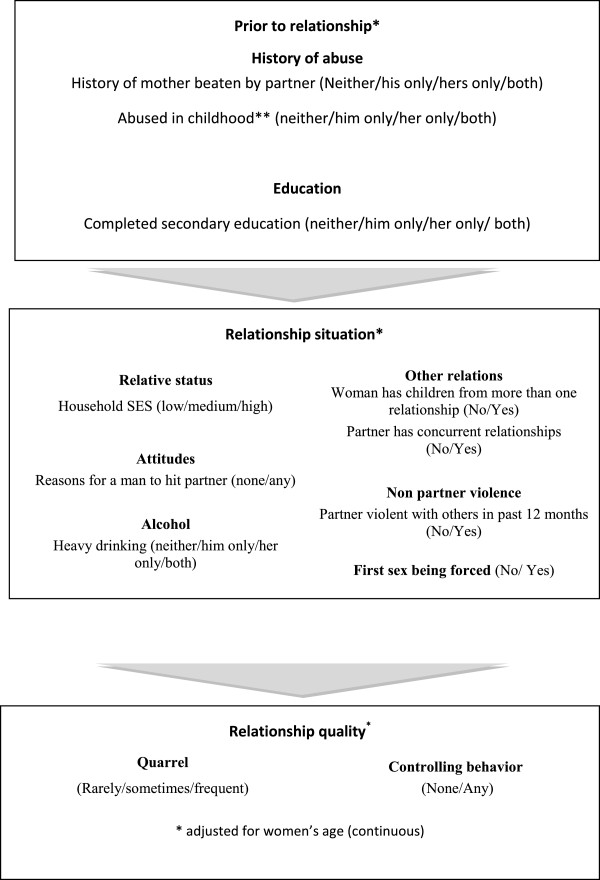
**Conceptual framework outlining variable description.**

IPV is a consequence of a complex combination of individual, relationship and societal factors. To investigate the factors associated with gender-based violence, a relationship approach framework was used that separately considers factors that existed prior to the relationship, factors that characterize the current relationship and factors relating to the relationship quality (Figure 1). A similar conceptual framework was used in an earlier paper that analyzed the factors associated with IPV among all age groups [29] and was adapted for this analysis, by adding the relationship quality level. This framework considers factors that operate at different levels, while separating out factors that occurred in both the woman’s and her partner’s past, factors that influence their current relationship and factors indicative of the overall relationship quality. Variables that were included in the analysis that occurred prior to the relationship include whether the woman’s or her partner’s mother was beaten or both, whether the partner was physically and the woman sexually abused during childhood or both and whether the woman and her partners have a secondary education or both. Variables investigated at the current relationship level include the woman’s household status relative to her partner, her attitude towards IPV, women’s and their partner’s heavy drinking and woman’s marital status. It also examined the effect of a woman having children from more than one relationship, the partner having concurrent relationships or being involved in fights outside the home in the last year and if the woman’s first sex was wanted, unwanted or forced. At the relationship quality level, couple’s frequency of quarrel and existing controlling behavior was examined. Controlling behavior is measured by a score created out of the seven questions capturing controlling behaviors, ranging from trying to keep the woman from seeing her friend and family to expecting the woman to ask for permission to seek health care. The relationship quality level was analyzed separately as frequency of quarrel and controlling behavious are often seen as forms of IPV and therefore conceptually different from the risk factors analyzed at the current relationship level, which are clearly acknowledged as risk and protective factors of IPV.

### Statistical analysis

The analysis was limited to “ever-partnered” women between 15 and 24 years old to capture those women who were going through the biological changes and social-role transitions that define adolescence and young adulthood [24]. The definition of “ever-partnered” used in this study was based on the cultural and legal definitions in the respective countries. It captured women who stated that they were ever married, ever lived with a man or were currently with a regular sexual partner in all countries. In Namibia and Peru it also included having ever been with a regular sexual partner. In Bangladesh, the definition was restricted to ever-married women. The proportion of ever-partnered women among 15 to 24 varied from 30 percent in Thailand city to 70 percent in Namibia (see Table 1).

**Table 1:** **Proportion of ever-partnered women aged 15 to 24, who experienced physical and/or sexual intimate partner violence during their lifetime and in the last 12 months, by site**

	% of 15–24 year olds who are currently or ever-partnered	Total n of 15-24 year old ever-partner women	% of ever-partnered 15–24 year olds with >1 partner ever	% IPV among ever-partnered 15–24 years old	% IPV last 12 months among ever-partnered 15–24 years old
Bangladesh city	65.9%	388	1.0%	56.7%	39.9%
Bangladesh province	62.9%	321	2.5%	53.3%	36.4%
Brazil city	56.8%	188	5.3%	22.9%	14.4%
Brazil province	54.7%	267	9.7%	34.8%	23.2%
Ethiopia province	35.3%	399	3.8%	65.7%	57.4%
Namibia city	75.0%	320	5.0%	37.5%	26.2%
Peru city	44.8%	208	1.9%	51.0%	31.1%
Peru province	51.5%	282	2.8%	66.0%	45.0%
Samoa	35.1%	181	4.4%	47.5%	33.5%
Serbia & Montenegro city	55.1%	199	1.5%	19.1%	7.50%
Thailand city	30.6%	124	10.4%	45.2%	32.8%
Thailand province	38.9%	112	10.5%	51.8%	32.5%
U. Rep. Tanzania city	58.2%	421	7.2%	36.6%	27.4%
U. Rep. Tanzania province	69.0%	368	3.2%	47.6%	33.2%

The prevalence of IPV in the last 12 months was calculated for ever-partnered women across all age groups, namely 15 to 24, 25 to 34 and 35 to 49 for each site. Among women aged 15 to 24 the prevalence of IPV in the last 12 months was also compared to the lifetime prevalence.

Descriptive statistics were produced for all associated factors for IPV outlined in the conceptual framework among ever-partnered women aged 15 to 24.

Multivariable logistic regressions were conducted for each site on factors associated with the lifetime experience of IPV for ever-partnered women aged 15 to 24. Women who experienced IPV in their current or last relationship were compared to women who did not report IPV.Women who only experienced IPV in a prior but not their current relationship were excluded from the analysis. This was done to avoid diluting associations and to prevent women’s and their partner’s characteristics to be treated as abused if women were abused in a prior but not their current relationship

The first model investigated factors occurring prior to the relationship, including women’s and their partner’s history of childhood abuse and educational attainment, controlling for women’s current age. The second model examined factors relating to women’s current relationship situation, including household status, attitudes towards violence, alcohol use, marital status, other relations and their first sexual experience. This model controlled for women’s age and educational differences. In the third model factors capturing relationship quality, such as frequent quarrelling and controlling behavior are examined for their association with IPV, controlling for women’s age.

All analyses were conducted using STATA statistical analysis software. Statistical significance is considered at the 5% level. Missing values are marginal. In the few cases, when more than 5% of data were absent, a special category was generated and controlled for (data not shown).

## Results

### Prevalence

The proportion of young women aged 15 to 24, who ever experienced IPV is high across all study sites. In many sites, the prevalence is around 50 percent or higher. The lowest is 19 percent in the urban site in Serbia, the highest is 66 percent in the site in rural Peru. The proportion of women experiencing IPV in the last 12 months is also high among women aged 15 to 24, with prevalence rates ranging from seven percent in the urban site in Serbia to 57 percent in the rural site in Ethiopia. Details for other countries are displayed in Table 1.

**Figure 2:**
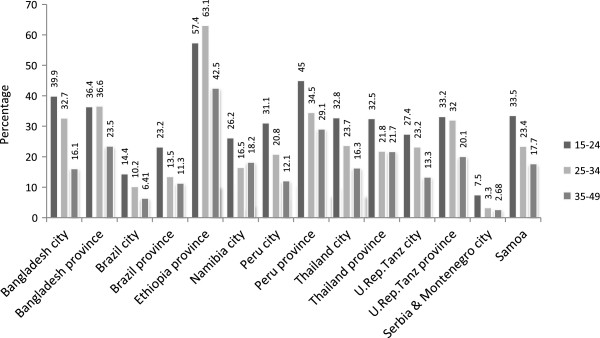
**Proportion of women experiencing intimate partner violence during the last 12 months among all ever-partnered women according to their age, by site.**

In almost all countries, women aged 15 to 24 reported higher levels of IPV during the last 12 months than women above the age of 24, with the exception of the rural site in Ethiopia where 25 to 34 year old women reported higher levels of IPV (see Figure 2).

### Factors associated with IPV among young women

#### Prior to relationship

Among the four risk factors of experiencing IPV among young women that occurred prior to their current relationship, witnessing one’s mother being beaten by her partner was associated with IPV across nearly all sites. The prevalence of women who stated that both her and her partner’s mother were beaten by their father ranged from one percent in the urban site in Namibia to 19 percent in the rural Peruvian site. Significant associations between experiencing IPV and witnessing of one’s own or one’s partner’s mother being hit by a partner were found in all sites except in urban Brazil, urban Serbia and urban Thailand. The association was strongest when the woman’s mother or both the woman’s and her partner’s mother were hit. Only the partner witnessing his mother being abused and not the woman witnessing her mother being abused was less strongly associated with IPV, except in urban and rural Bangladesh, urban Peru and rural Tanzania.

Experiencing sexual abuse during childhood (before age 15) was only significantly associated with IPV among adolescents in urban Bangladesh, while having a partner who was physically abused as a child was only significantly associated with IPV in rural Brazil and urban Peru. Secondary education was protective against IPV if both the woman and her partner had it in urban Bangladesh and in urban Tanzania. In urban Bangladesh, it was also protective if only the partner had secondary education. Details on the prevalence of ‘prior relationship’ risk factors and their associations with IPV are shown in Table 2.

**Table 2:** AOR* (95% CI) and 95% CIs for associations between ‘prior to relationship’ variables and ever experiencing intimate partner violence among ever-partnered women aged 15-24

			BGD urban		BGD rural		BRA urban		BRA rural		ETH rural		NAM urban		PER urban
			n = 373		n = 298		n = 186		n = 262		n = 330		n = 283		n = 195
		%	AOR(95% CI)	%	AOR(95% CI)	%	AOR(95% CI)	%	AOR(95% CI)	%	AOR(95% CI)	%	AOR(95% CI)	%	AOR(95% CI)
History of mother beaten by partner	Neither	74		79		67		68		77		76		41	
	His only	9	6.38^***^	11	4.73^**^	15	1.32	13	1.99	3	0.88	2	0.87	17	2.77^*^
			[2.29,17.79]		[1.65,13.60]		[0.41,4.26]		[0.82,4.82]		[0.19,4.17]		[0.15,4.98]		[1.17,6.60]
	Hers only	14	3.42^**^	8	2.18	15	2.11	14	1.48	16	2.35^*^	21	3.65^***^	26	3.03^*^
			[1.61,7.27]		[0.89,5.36]		[0.69,6.42]		[0.62,3.51]		[1.21,4.58]		[2.02,6.60]		[1.38,6.66]
	Both	3	2.03	2	5.30	3	5.53	5	4.41^*^	4	1.21	1	2.52	16	4.75^***^
			[0.34,12.35]		[0.56,50.35]		[0.90,33.95]		[1.24,15.69]		[0.26,5.60]		[0.32,19.73]		[1.89,11.92]
Women’s child sexual abuse	No	83		88		91		92		98		92		73	
	Yes	17	8.92^***^	12	.	9	0.92	8	1.70	2	4.43	8	2.48	27	1.99
			[3.61,22.05]		[3.61,22.05]		[0.22,3.87]		[0.58,4.96]		[0.49,40.00]		[0.97,6.33]		[0.98,4.05]
Partner physically beaten as a child	No	84		91		80		79		96		92		65	
	Yes	16	1.35	9	2.30	20	2.31	21	3.24^**^	4	3.46	8	0.97	35	1.17
			[0.67,2.74]		[0.91,5.83]		[0.84,6.32]		[1.56,6.76]		[0.56,21.38]		[0.73,1.29]		[0.80,1.71]
Completed secondary education	Neither	24		42		32		68		94		10		2	
	Him only	15	0.41^*^	10	0.45	13	0.94	8	2.29	4	0.91	9	1.03	5	0.46
			[0.19,0.90]		[0.17,1.19]		[0.26,3.37]		[0.82,6.42]		[0.29,2.83]		[0.32,3.33]		[0.03,7.47]
	Her only	7	0.48	12	1.45	14	0.95	10	0.59	1	0.19	6	2.35	1	.
			[0.17,1.30]		[0.64,3.24]		[0.26,3.49]		[0.21,1.72]		[0.02,2.31]		[0.64,8.61]		-
	Both	54	0.32^***^	36	0.96	41	0.88	14	0.71	1	2.07	75	1.19	92	0.76
			[0.17,0.59]		[0.55,1.68]		[0.33,2.37]		[0.26,1.92]		[0.22,19.40]		[0.50,2.80]		[0.06,9.37]
			**PER rural**		**SMA**		**SRB urban**		**THA urban**		**THA rural**		**TAZ urban**		**TZA Rural**
			**n = 278**		**n = 180**		**n = 153**		**n = 112**		**n = 107**		**n = 386**		**n = 339**
		**%**	**AOR(95% CI)**	**%**	**AOR(95% CI)**	**%**	**AOR(95% CI)**	**%**	**AOR(95% CI)**	**%**	**AOR(95% CI)**	**%**	**AOR(95% CI)**	**%**	**AOR(95% CI)**
History of mother beaten by partner	Neither	39		47		86		67		52		70		45	
	His only	10	1.96	2	0.75	3	1.44	5	0.92	9	2.02	3	1.56	6	2.59^*^
			[0.77,4.94]		[0.06,8.78]		[0.15,14.06]		[0.14,6.17]		[0.44,9.17]		[0.46,5.29]		[1.03,6.54]
	Hers only	32	3.00^***^	45	1.08	10	2.29	23	0.88	29	2.94^*^	24	2.30^**^	37	2.63^***^
			[1.56,5.78]		[0.55,2.13]		[0.57,9.15]		[0.34,2.24]		[1.08,8.03]		[1.40,3.80]		[1.59,4.33]
	Both	19	3.51^**^	6	14.03^*^	1	.	5	2.68	10	4.99^*^	3	3.45^*^	12	2.84^**^
			[1.52,8.11]		[1.55,127.32]		-		[0.43,16.93]		[1.04,23.84]		[1.04,11.39]		[1.39,5.81]
Woman’s child sexual abuse	No	91		97		98		89		88		93		96	
	Yes	9	0.75	3	2.91	2	.	11	1.06	12	1.52	7	1.36	4	0.86
			[0.29,1.98]		[0.26,32.53]		-		[0.30,3.76]		[0.38,6.11]		[0.57,3.28]		[0.27,2.67]
Partner physically beaten as a child	No	67		90		95		86		78		95		95	
	Yes	33	1.10	10	1.83	5	0.93	14	2.75	22	1.92	5	2.05	5	2.07
			[0.58,2.08]		[0.56,5.92]		[0.49,1.74]		[0.82,9.23]		[0.63,5.87]		[0.77,5.41]		[0.72,5.89]
Completed secondary education	Neither	20		11		1		21		32		58		77	
	Him only	31	1.07	3	0.31	1	7.64	13	1.43	8	0.84	23	0.92	15	0.78
			[0.50,2.26]		[0.03,3.55]		[0.44,133.99]		[0.38,5.48]		[0.16,4.46]		[0.54,1.56]		[0.40,1.53]
	Her only	3	0.54	5	0.28	3	1.80	10	0.66	16	0.56	2	0.87	3	0.41
			[0.12,2.46]		[0.04,2.09]		[0.18,17.81]		[0.14,3.11]		[0.16,2.00]		[0.21,3.57]		[0.10,1.64]
	Both	46	0.84	81	0.95	95	.	56	1.32	44	0.60	17	0.44^*^	5	0.83
			[0.42,1.70]		[0.35,2.59]		--		[0.49,3.53]		[0.23,1.59]		[0.23,0.86]		[0.29,2.31]

*Adjusted for age (continuous), (data not shown).

*** = p-value<0.001, **= p-value <0.01, *= p-value<0.05.

#### Relationship situation

Clear patterns also emerged when investigating the associations between factors related to the ‘current situation’ and IPV across the different sites. As displayed in Table 3, the factors that were most commonly associated with IPV among adolescents and young women were heavy drinking by the partner, partner having concurrent relationships and partner being involved in fights with other men.

**Table 3:** AOR* (95% CI) and 95% CIs for associations between ‘relationship situation’ variables and ever experiencing intimate partner violence among ever-partnered women aged 15-24

			BGD urban		BGD rural		BRA urban		BRA rural		ETH rural		NAM urban		PER urban
		%	AOR(95% CI)	%	AOR(95% CI)	%	AOR(95% CI)	%	AOR(95% CI)	%	AOR(95% CI)	%	AOR(95% CI)	%	AOR(95% CI)
Reasons for a man to hit partner	None	42		21		81		60		7		76		60	
	Any	58	1.68	79	0.77	19	2.13	40	2.01	93	2.98^*^	24	1.72	40	1.79
			[1.00,2.83]		[0.37,1.60]		[0.75,6.08]		[0.98,4.13]		[1.20,7.42]		[0.91,3.24]		[0.90,3.53]
Heavy drinking	Neither	99		100		74		64		86		54		71	
	He	1	2.44	0	.	7	9.84^**^	21	1.87	5	2.15	25	1.94^*^	19	0.85
			[0.16,37.19]		[0.16,37.19]		[2.18,44.34]		[0.82,4.25]		[0.62,7.46]		[1.00,3.77]		[0.34,2.10]
	She	0	.	0	.	17	1.10	9	2.01	5	0.66	12	1.49	8	0.68
			-		-		[0.29,4.08]		[0.55,7.32]		[0.24,1.81]		[0.63,3.55]		[0.20,2.36]
	Both	0	.	0	.	2	.	6	9.76^**^	4	6.01	9	1.16	2	2.81
			-		-		-		[2.39,39.79]		[0.67,53.53]		[0.45,2.98]		[0.23,34.88]
Partner has concurrent relationship (missing not shown)	No	94		95		85		73		91		48		79	
	Yes	4	7.31	2	.	10	1.19	19	2.60^*^	9	0.87	29	2.53^**^	15	2.17
			[0.68,79.16]		[0.68,79.16]		[0.26,5.52]		[1.16,5.83]		[0.39,1.96]		[1.37,4.69]		[0.80,5.91]
Partner involved in fight with man	No	95		96		89		88		92		88		84	
	Yes	5	7.74^*^	4	4.63	11	3.84^*^	12	5.04^**^	8	1.79	12	2.08	16	2.78^*^
			[1.46,40.94]		[0.37,58.41]		[1.05,14.11]		[1.89,13.45]		[0.68,4.68]		[0.90,4.82]		[1.06,7.34]
Woman’s first sexual intercourse	Wanted	62		54		74		70		23		46		52	
	Unwanted	15	0.81	18	2.34^*^	25	1.47	27	1.41	59	1.78^*^	48	2.07^*^	44	1.98^*^
			[0.41,1.60]		[1.20,4.57]		[0.54,3.97]		[0.65,3.03]		[1.02,3.12]		[1.16,3.67]		[1.02,3.86]
	Forced	23	106.21^***^	28	.	1	.	3	0.60	18	3.59^**^	6	2.77	4	6.21
			[13.99,806.63]		-		-		[0.08,4.61]		[1.63,7.89]		[0.88,8.70]		[0.99,38.90]
			**PER rural**		**SMA**		**SRB urban**		**THA urban**		**THA rural**		**TAZ urban**		**TZA Rural**
		**%**	**AOR(95% CI)**	**%**	**AOR(95% CI)**	**%**	**AOR(95% CI)**	**%**	**AOR(95% CI)**	**%**	**AOR(95% CI)**	**%**	**AOR(95% CI)**	**%**	**AOR(95% CI)**
Reasons for a man to hit partner	None	17		22		93		44		30		28		27	
	Any	83	2.31	78	2.77^*^	7	0.18	56	0.57	70	0.53	72	1.54	73	2.26^**^
			[0.97,5.50]		[1.14,6.73]		[0.02,2.13]		[0.23,1.43]		[0.17,1.65]		[0.87,2.71]		[1.29,3.95]
Heavy drinking	Neither	57		70		71		65		65		75		72	
	He	25	2.63^*^	29	2.25^*^	3	.	18	2.30	25	3.17	10	1.28	8	3.74^**^
			[1.16,5.93]		[1.03,4.91]		-		[0.65,8.13]		[0.92,10.90]		[0.59,2.79]		[1.47,9.50]
	She	10	0.98	0	.	23	2.74	9	1.06	6	1.00	7	3.21^**^	14	2.14^*^
			[0.37,2.60]		-		[0.78,9.66]		[0.22,5.10]		[0.13,8.01]		[1.40,7.32]		[1.09,4.17]
	Both	8	6.05^*^	1	0.63	3	1.54	8	1.99	4	.	6	1.02	6	11.19^**^
			[1.22,30.13]		[0.03,12.69]		[0.11,20.72]		[0.32,12.28]		[0.32,12.28]		[0.41,2.56]		[2.15,58.13]
Partner has concurrent relationship (missing not shown)	No	72		89		93		77		78		56		73	
	Yes	18	3.07^*^	9	0.83	4	2.34	8	3.98	17	9.91^*^	13	2.49^**^	12	2.08
			[1.14,8.29]		[0.23,3.06]		[0.19,29.48]		[0.69,22.85]		[1.57,62.41]		[1.28,4.85]		[0.94,4.60]
Partner involved in fight with man	No	80		81		77		82		78		96		97	
	Yes	20	1.35	19	3.89^**^	23	4.85^**^	18	7.13^**^	22	2.85	4	0.75	3	9.25
			[0.57,3.21]		[1.53,9.92]		[1.55,15.13]		[1.94,26.28]		[0.79,10.24]		[0.24,2.35]		[0.84,101.75]
Woman’s first sexual intercourse	Wanted	42		82		94		64		59		62		65	
	Unwanted	37	3.91^***^	12	1.89	5	.	33	1.66	30	2.44	24	1.36	15	0.95
			[1.96,7.79]		[0.65,5.52]		-		[0.60,4.64]		[0.68,8.69]		[0.78,2.36]		[0.49,1.87]
	Forced	21	4.98^***^	6	2.16	1	.	3	1.14	11	2.27	14	2.45^**^	20	2.46^**^
			[2.03,12.23]		[0.50,9.25]		-		[0.08,16.08]		[0.43,11.97]		[1.25,4.82]		[1.33,4.56]

*Adjusted for age (continuous), educational differences, having a child with more than one biological father, socio-economic and marital status (data not shown due to lack of significance in most sites).

*** = p-value<0.001, **= p-value <0.01, *= p-value<0.05.

Heavy drinking was significantly associated with IPV among young women across a number of sites, with stronger associations found when only the partner or both the woman and her partner were drinking. A significant association between heavy drinking of the partner or both the woman and her partner and experiences of IPV was found in urban Brazil, rural Brazil, urban Namibia, rural Peru, Samoa, and urban and rural Tanzania.

The prevalence of concurrent partnerships of the partner as reported by women varied strongly across the different sites, with two percent of women aged 15 to 24 in rural Bangladesh reporting that their partner has a concurrent relationship and up to 29 percent in urban Namibia. Significant associations between partner’s concurrent relationships and IPV were found in rural Brazil, urban Namibia, rural Peru, rural Thailand, and urban Tanzania.

Between four and 19 percent of adolescents and young women reported that their partner was involved in fights with other men. This was strongly associated with IPV experiences among adolescents and young women in sites in rural Bangladesh, urban and rural Brazil, urban Namibia, Samoa, urban Serbia, urban Thailand and rural Tanzania.

Having a high socio-economic status compared to a low one was protective against IPV for adolescents and young women in Samoa (OR: 0.32; CI: 0.11,0.97), while it was not significant in the other sites. Not being married was also not a significant correlate with IPV among young women, except for Namibia, where it increased women’s likelihood of IPV (OR: 3.84; CI: 1.02,14.56) and Samoa, where it reduced women’s likelihood of IPV (OR: 0.42; CI: 0.20,0.91). Interestingly, having more than one child from different fathers was negatively associated with IPV among adolescent and young women in sites in rural Brazil (OR: 0.30; CI: 0.12,39.79), and urban Serbia (OR: 0.12; CI: 0.02,0.93).

Women’s experience of first sex being forced ranged between one percent in urban Brazil and 28 percent in rural Bangladesh. In addition, five percent in urban Serbia and up to 50 percent in rural Ethiopia reported that their first sexual intercourse was unwanted, albeit not forced. First sexual intercourse being forced was significantly associated with IPV among young women in urban Bangladesh, rural Ethiopia, rural Peru, and urban and rural Tanzania; having had an unwanted first sexual intercourse was significantly associated in rural Bangladesh, rural Ethiopia, urban Namibia and urban and rural Peru. The wide confidence interval in urban Bangladesh can be explained by the fact that only one out of 85 women who reported their first intercourse as being forced did not report IPV, while in rural Bangladesh all women who reported forced first sexual intercourse experienced IPV.

Acceptance of wife beating varied substantially across countries, with seven percent of adolescents and young women in a site in urban Serbia stating that a man is justified to beat his wife under certain conditions, compared to 93 percent in rural Ethiopia. Acceptance of wife beating was significantly associated with a higher likelihood of IPV experiences among young women in rural Ethiopia, Samoa, urban Tanzania and rural Tanzania.

#### Relationship quality

Adolescents and young women’s relationship quality, captured by measures of controlling behavior and frequency of quarrelling, is strongly associated with experiences of IPV across all sites. Adolescent and young women across all sites reported high levels of controlling behaviors, with the prevalence of highly controlling behavior ranging from five percent in urban Serbia to 55 percent in urban Tanzania. Table 4 also shows that high levels of controlling behavior were significantly associated with IPV in all sites except rural Ethiopia and rural Thailand. Medium levels of controlling behavior were associated with IPV among adolescents and young women in urban Bangladesh, rural Brazil, rural Ethiopia, urban Namibia, rural Peru, and urban Serbia.

**Table 4:** **AOR (95% CI)s and 95% CIs for associations between for ‘relationship quality’ variables and ever experiencing intimate partner violence among ever-partnered women aged 15-24**

			BGD urban		BGD rural		BRA urban		BRA rural		ETH rural		NAM urban		PER urban
		%	AOR(95% CI)	%	AOR(95% CI)	%	AOR(95% CI)	%	AOR(95% CI)	%	AOR(95% CI)	%	AOR(95% CI)	%	AOR(95% CI)
Controlling behavior	No control	44		27		33		31		36		36		19	
	Medium	38	1.91^**^	51	1.08	42	1.55	37	4.76^**^	45	2.03^**^	37	2.34^*^	46	1.66
			[1.19,3.06]		[0.63,1.86]		[0.49,4.90]		[1.78,12.76]		[1.21,3.40]		[1.21,4.50]		[0.70,3.89]
	High	18	10.22^***^	22	2.02^*^	25	3.82^*^	32	9.99^***^	19	1.40	27	5.76^***^	35	5.15^***^
			[4.23,24.69]		[1.02,3.98]		[1.19,12.27]		[3.68,27.10]		[0.72,2.73]		[2.88,11.52]		[2.00,13.24]
Frequency of quarrel	Rarely	61		75		48		44		66		46		42	
	Sometimes	33	1.79^*^	23	2.15^**^	41	1.45	49	1.16	27	6.95^***^	44	1.89^*^	43	1.33
			[1.10,2.91]		[1.21,3.81]		[0.56,3.75]		[0.59,2.30]		[3.49,13.82]		[1.09,3.30]		[0.69,2.55]
	Often	6	4.39	2	4.76	11	5.00^**^	7	5.63^**^	7	-	10	5.09^***^	15	1.84
			[0.91,21.22]		[0.54,42.18]		[1.50,16.63]		[1.65,19.19]		-		[2.02,12.83]		[0.69,4.93]
			**PER rural**		**SMA**		**SRB urban**		**THA urban**		**THA rural**		**TAZ urban**		**TZA Rural**
		**%**	**AOR(95% CI)**	**%**	**AOR(95% CI)**	**%**	**AOR(95% CI)**	**%**	**AOR(95% CI)**	**%**	**AOR(95% CI)**	**%**	**AOR(95% CI)**	%	**AOR(95% CI)**
Controlling behavior	No control	18		19		66		24		25		7		22	
	Medium	39	2.39^*^	47	2.11	29	3.17^*^	47	2.57	39	1.07	38	2.29	39	1.41
			[1.12,5.13]		[0.77,5.78]		[1.22,8.28]		[0.86,7.73]		[0.35,3.32]		[0.71,7.34]		[0.74,2.70]
	High	43	10.25^***^	34	6.35^***^	5	10.24^**^	29	10.48^***^	36	1.59	55	4.64^**^	39	4.06^***^
			[4.38,24.01]		[2.20,18.33]		[2.06,51.02]		[2.99,36.71]		[0.51,4.98]		[1.50,14.38]		[2.12,7.78]
Frequency of quarrel	Rarely	50		71		63		61		56		55		62	
	Sometimes	42	4.28^***^	15	5.34^**^	30	2.35	31	2.89^*^	27	2.71^*^	37	3.33^***^	29	3.13^***^
			[2.28,8.02]		[1.86,15.28]		[0.93,5.93]		[1.18,7.04]		[1.03,7.14]		[2.06,5.39]		[1.88,5.24]
	Often	8	2.34	14	3.46^*^	7	0.79	8	1.05	17	6.93^**^	8	5.60^***^	9	6.07^***^
			[0.69,7.98]		[1.26,9.51]		[0.14,4.60]		[0.24,4.62]		[1.85,26.02]		[2.52,12.41]		[2.32,15.88]

*Adjusted for women’s age (continuous), (data not shown).

*** = p-value<0.001, **= p-value <0.01, *= p-value<0.05.

Between two percent of adolescents and young women in urban Bangladesh and 17 percent in rural Thailand stated that they quarrel often with their partner. Quarrelling sometimes or often with one’s partner was significantly associated with IPV in all sites, except urban Peru and urban Serbia.

## Discussion

This study is the first to examine IPV among adolescents and young women across different countries and settings, using population based comparable survey data. It shows that adolescent and young women are most at risk of experiencing physical and sexual IPV among women of reproductive age, with prevalence rates ranging from eight to 57 percent for physical and sexual IPV in the last 12 months, and their prevalence rates being higher than among older women in most countries.

There are several reasons for the decrease of IPV with age. One possible explanation is that the decrease is part of the general trend that criminal activity reduces with age [31], another is that couples who form early unions are more likely to face relationship stressors that can lead to IPV, such as early pregnancies, employment instability, and financial difficulties [32].

Factors associated with IPV that emerged as significant across several sites in this study as well as the 2011 study of all women of reproductive age [29] included witnessing violence against the mother, partner’s heavy drinking and partner’s involvement in fights. Factors that emerged as significant in this study that have not been measured in the 2011 study were women’s first sexual experience being unwanted or forced, frequent quarrels with partner and partner’s controlling behavior. In the 2011 study, education, high socio-economic status and formal marriage were important protective factors for IPV and attitudes favoring wife beating were important risk factors for IPV [29]. In this study however the evidence for the strength of these factors was not uniform across countries.

Witnessing IPV at home during childhood is believed to increase the likelihood of experiencing IPV through multiple mechanisms. Children might learn to model the behavior of their parents, are taught that violent behavior is unpunished and a strategies to gain authority and it harms the child emotionally and developmentally. Linked with the lack of knowledge on non-violent coping strategies, this can result in later behavioral problems, including violent behavior [33]. The lack of constructive coping skills might also explain the consistent finding across sites of the association between quarrelling and controlling behavior with IPV in this study. These findings therefore highlight the importance of starting prevention efforts early to reduce the occurrence of IPV. Increased efforts are needed to reduce abuse in childhood through early childhood interventions such as home visitation programmes, supporting children who witnessed violence in their homes, parenting interventions to improve parent–child relationships and reduce negative parenting practices and other strategies have been outlined elsewhere [34, 35]. In addition, more focus needs to be put on the development of programmes increasing the ability of adolescents and young adults to improve their negotiation and interpersonal skills and to engage in non-controlling relationships.

The study’s findings on the association of alcohol usage and IPV is in line with scores of studies that have shown a strong and consistent association between IPV and alcohol usage among abusive men and among women experiencing IPV, including a recent systematic review [36]. Established pathways linking alcohol abuse and IPV include raising levels of aggression, misinterpretation of verbal or non-verbal cues, increased risk taking behavior and the fact that alcohol usage might be a source of argument in relationships [36]. IPV is therefore another important reason to tackle alcohol use among adolescents. In addition, existing programmes on alcohol abuse should also try to raise awareness of its connections with violence to reduce its effect on IPV [37].

Several limitations of the study have to be kept in mind when interpreting the results. First, the prevalence rates stated are likely to be conservative estimates because many women might choose not to reveal IPV, despite the measures taken to ensure safety, privacy and confidentiality so that women more accurately report IPV. Secondly, given the cross-sectional nature of this study, no conclusion can be drawn regarding the causal nature of observed relationships, with the exception of experiences in the woman’s childhood. Thirdly, questions about partners’ experiences or behaviors were based on respondents’ knowledge rather than self-report, making these variables less stable measures. Fourthly, the initial sample size calculations were made for women aged 15 to 49. Limiting the analysis to women aged 15 to 24 made it difficult to find significant associations in countries where the sample size among this age group was small. Initially, separate analyses were proposed to investigate the factors associated with IPV for women aged 15 to 19 and 20 to 24 years, but this was not possible due to too few women for meaningful analyses in these groups. The small sample size further limited the possibility to analyze physical and sexual IPV separately. In addition, we excluded women from the analysis who experienced IPV in a prior, but not their current relationship. As well as the restriction of the sample to ever-partnered women, which in most countries was defined as ever married or cohabiting, this might have excluded potentially violent, often short term, intimate relationships in which adolescents and young women might engage in, such as dating relationships, short-term boyfriends or casual sexual partners. Using an existing study dedicated to researching women of all reproductive ages also limited the inclusion of risk factors specific to this adolescent and young women, such as peer pressure, parental education, living with biological parents and school attendance [10, 12].

Despite these limitations, the WHO Multi-country study is one of the few surveys that collected internationally comparable quantitative data on IPV. This makes the study unique when investigating IPV against adolescents and young women, a topic for which few representative studies exist outside North America. It also highlights the need for more focused research on IPV among adolescents and young women using large-scale survey data. In order to understand the phenomena of IPV among adolescents and young women, this population needs to be oversampled in large population based surveys. At this stage, large-scale surveys do not include sufficient numbers of young women to allow in-depth investigations. Existing surveys targeting adolescents and young women should consider including questions on IPV, if they can meet the necessary ethical and safety standards needed for this kind of research [38]. More research is also needed on the sexual and reproductive health outcomes of IPV among adolescents and young women, especially in low and middle-income countries. Recent studies on reproductive coercion from the United States have shown the many ways in which IPV limits young women’s reproductive choices and sexual and reproductive health [21, 39], but little evidence exists outside the United States.

## Conclusion

The findings of this study have numerous implications for the promotion and safeguarding of adolescent and young women’s health. The high prevalence of IPV among adolescents and young women is alarming. Policies and services aimed at preventing and addressing IPV need to increase and existing programmes need to be tailored to reach adolescent and young women.

The known health effects of IPV, especially on women’s sexual and reproductive and mental health, further calls for the integration of strategies to address IPV, promote gender equality and foster equitable sexual relationships in programmes focusing on the promotion and safeguarding of sexual and reproductive health of adolescents and young women. In addition, tailored strategies should be incorporated into programs targeting young men, such as male circumcision programmes, or programs targeting young women, such as family planning programs. Given the strong link that previous studies have established between IPV and unplanned pregnancy, miscarriage and abortion [40, 41], it is crucial for sexual and reproductive health programs to address IPV.

Adolescence and early adulthood is an important period in laying the foundation for healthy and stable relationships, and women’s health and well-being overall. Ensuring that adolescents and young women enjoy relationships free of violence is an important investment in their future.
